# Bioassay-Guided Isolation of Piplartine from *Piper purusanum* Yunck (Piperaceae) and Evaluation of Its Toxicity Against *Aedes aegypti* Linnaeus, 1762, *Anopheles darlingi* Root, 1926 (Culicidae), and Non-Target Animals

**DOI:** 10.3390/plants14050774

**Published:** 2025-03-03

**Authors:** André Correa de Oliveira, Felipe Moura Araujo da Silva, Ingrity Suelen de Sá, Brenda Reis Coelho Leocadio, Suelen Costa Lima, Maria Luiza Lima da Costa, Rosemary Aparecida Roque, Rita de Cássia Saraiva Nunomura

**Affiliations:** 1 Laboratório de Controle Biológico e Biotecnologia da Malária e da Dengue, Coordenação Sociedade, Ambiente e Saúde, Instituto Nacional de Pesquisas da Amazônia, Manaus, Amazonas 69067-375, Brazilluiza3646@hotmail.com (M.L.L.d.C.);; 2 Programa de Pós-Graduação em Ciências Biológicas (Entomologia), Instituto Nacional de Pesquisas da Amazônia, Manaus, Amazonas 69067-375, Brazil; 3 Laboratório de Abertura de Amostras e Ensaios Químicos, Central Analítica, Centro de Apoio Multidisciplinar, Universidade Federal do Amazonas, Manaus, Amazonas 69080-900, Brazil; ingrity.scosta@gmail.com (I.S.d.S.); brenda.rleocadio@gmail.com (B.R.C.L.);; 4 Laboratório de Cromatografia e Espectrometria de Massas, Central Analítica, Centro de Apoio Multidisciplinar, Universidade Federal do Amazonas, Manaus, Amazonas 69067-375, Brazil

**Keywords:** compound, dengue, malaria, enzymes, eco-friendly, plant

## Abstract

*Aedes aegypti* and *Anopheles darlingi* are the primary vectors of dengue and malaria in Brazil. Natural products are currently regarded as promising alternatives for their control, offering environmentally friendly solutions for larval management due to their low toxicity to non-target organisms. Thus, Piplartine, isolated for the first time from *Piper purusanum*, exhibited larvicidal activity against *Ae. aegypti* and *An. darlingi* (LC_50_ of 14.56 and 26.44 μg/mL), occasioned by the overproduction of reactive oxygen and nitrogen species (66.67 ± 7% and 86.33 ± 6%). Furthermore, piplartine enhanced the activity of key detoxifying enzymes, including catalase (87.00 ± 9 and 94.67 ± 9 μmol of H_2_O_2_ consumed per minute per mg of protein), glutathione S-transferase (76.00 ± 1 and 134.00 ± 1 μmol/min/mg), mixed-function oxidase (26.67 ± 5 and 55.00 ± 1 nmol cti mg⁻¹ protein), α-esterase, and β-esterase (27.67 ± 7 to 46.33 ± 1 nmol cti mg⁻¹ protein). In contrast, piplartine inhibited acetylcholinesterase activity (43.33 ± 7 and 48.00 ± 2 μmol/min/mg) compared to the negative control DMSO (87.33 ± 1 and 146.30 ± 3 μmol/min/mg). It is important to highlight that piplartine showed no lethal effects on non-target aquatic insects, with 100% survival observed at a concentration of 264.4 μg/mL. In contrast, α-cypermethrin demonstrated acute and rapid toxicity to non-target organisms, with only 9.1% survival. These findings highlight piplartine as a promising larvicide with selective toxicity and low environmental impact, suitable for integrated larval management strategies.

## 1. Introduction

Dengue, Zika, and chikungunya are arboviral diseases transmitted by the dengue virus (DENV), which has four serotypes (DENV-1 to DENV-4), the Zika virus (ZIKV), both belonging to the *Flavivirus* genus (Flaviviridae family), and the chikungunya virus (CHIKV), classified under the *Alphavirus* genus (Togaviridae family) [1].

As of 19 December 2024, the Americas reported 12.78 million suspected dengue cases, with 6.78 million confirmed and 7822 fatalities (Pan American Health Organization (PAHO) [2]. The PAHO data also report 225,094 confirmed cases of chikungunya, with 211 fatalities, as well as 2048 cases of Zika with one fatality during the same period [3]. Brazil accounted for 53% of all arbovirus cases, with 6.89 million infections, including 6.62 million dengue cases (5959 deaths), 6081 Zika cases (no deaths), and 266,241 chikungunya cases (210 deaths) [2].

Another disease of significant epidemiological importance in the Americas is malaria, which Brazil, Venezuela, and Bolivia account for 73.0% of the cases [4]. In Brazil, malaria is primarily caused by the etiologic agents *Plasmodium vivax* and *P. falciparum* (Plasmodiidae), which are responsible for 82.7% and 13.3% of the 128,214 cases reported in the country in 2024, respectively [5].

The high incidence of malaria in Brazil is influenced by environmental and socioeconomic conditions that favor the proliferation of the mosquito *Anopheles darlingi* Root, 1926 (Diptera: Culicidae), the primary vector of the disease, and increase the population exposure to this transmitter [6]. Similarly, dengue, Zika, and chikungunya present an alarming scenario fueled by the presence of *Aedes aegypti* Linnaeus, 1762 (Diptera: Culicidae), in urban environments [1]. These factors result in a heterogeneous and complex spatial distribution, with varying scenarios and risks of occurrence for both malaria and these arboviruses [5].

Another critical factor influencing the proliferation of *An. darlingi* and *Ae. aegypti* mosquitoes is their increasing resistance to synthetic insecticides, performed by the action of detoxifying enzymes that play a crucial role in the survival and maintenance of these species [6]. Mosquitoes possess enzymatic systems such as esterases, oxidases, mono-oxygenases, and others like glutathione S-transferases, which help break down and neutralize the harmful effects of insecticides such as organophosphates, carbamates, and pyrethroids [6].

These enzymes provide a defense mechanism that allows these species to withstand exposure to synthetic chemicals [7]. Furthermore, oxidative stress, induced by the accumulation of reactive oxygen nitrogen species (RONS) during insecticide exposure, whether natural or synthetic, triggers the activation of these detoxifying enzymes as part of the organism’s defense response [8]. This adaptive mechanism enables mosquitoes to mitigate the damaging effects of oxidative stress, promoting their survival [9].

The enhanced detoxifying enzyme activity and oxidative stress tolerance in resistant mosquitoes enable their survival under high insecticide pressure, sustaining populations and disease transmission [10]. Therefore, the resistance reduces the effectiveness of chemical control strategies, allowing vector populations to persist and spread in both rural and urban environments [11].

Currently, detoxifying enzymes involved in mosquito defense against insecticides and those associated with neurological processes, including acetylcholinesterase, have been extensively studied [8]. Furthermore, oxidative stress caused by RONS during insecticide exposure has garnered scientific interest due to its role in vector survival mechanisms, being investigated not only to understand resistance processes but also to develop innovative control strategies that can overcome the limitations of current approaches [7].

Another problem in applying synthetic insecticides in breeding sites poses considerable environmental risks due to their high toxicity to non-target species, including beneficial organisms in aquatic ecosystems [12]. Indeed, recent research has shown that α-cypermethrin (LC_50_ between 0.22 and 0.29 μg/mL) [13] and temephos (LC_50_ between 4.85 and 5.82 μg/mL) [14] exhibit significantly higher toxicity against non-target species like *Toxorhynchites* sp. Theobald, 1901 (Diptera: Culicidae), *Anisops* sp. Spinola, 1837 (Hemiptera: Notonectidae), *Gambusia* sp. Poey, 1854 (Cyprinodontiformes: Poeciliidae), and *Diplonychus* sp. Leach, 1815 (Hemiptera: Belostomatidae).

Considering the diversity and ecological importance of these species in aquatic environments, as they play essential roles as predators, decomposers, and prey for other animals [15], it is imperative to adopt more sustainable and targeted mosquito control measures to mitigate or reduce the toxic effects on non-target organisms and the environment [16].

Due to growing concerns about environmental safety, insecticides based on natural bioactive compounds, particularly from plants, have become an effective alternative for controlling a wide range of insects with low toxicity to non-target organisms [17], providing a more sustainable solution for managing pests and disease vectors [18].

Plant-derived compounds used as insecticides are highly toxic to target insects specific to these organisms while being harmless to beneficial species and having a lower environmental impact [19]. Recent studies have shown that natural compounds extracted from plants exhibit biological activity, acting selectively on target organisms while preserving beneficial and essential species in aquatic ecosystems [20,21].

Insecticide compounds, such as amides, in arthropods primarily cause symptoms such as hyperactivity, hyper-excitation, rapid knockdown, and immobilization [22]. Several amides [20,21] can directly or indirectly affect a wide range of targets, including insect proteins, receptors, ion channels, neurotransmitter systems, and enzymes involved in signal transduction pathways, which act as agonists or antagonists, disrupting normal neural functions, leading to paralysis, convulsions, and eventual death.

Additionally, according to Rattan [23] and Ganesan et al. [24], some amides can also induce the overproduction of RONS, causing oxidative stress and cellular damage, as well as alterations in the activity of defense enzymes, including catalase (CAT), glutathione S-transferase (GST), thiols, esterases, and mixed-function oxidases (MFOs). Common targets of plant-derived compounds include acetylcholinesterase (AChE).

Several studies have reported the biological activity of amides derived from various plants, including those of the genus *Piper* (Piperaceae) against Culicidae. For example, pellitorine (from 0.4 to 5 μg/mL) [25,26,27], lansiumamide B (at 0.45 μg/mL) [28], piperine and piperanine (at 0.27 and 0.37 μg/mL) [29], piplartine (at 50 μg/mL) [30], n-isobutil-2*E*,6*Z*,8*E*-decatrienamide, n-isobutil-2*E*-decenamide, and n-isobutil-decanamide (from 4.24 to 50 μg/mL) [31], guineensine, retrofractamide A, and pipercide (from 1.5 to 3.2 μg/mL) [26], wuchuyuamide I (at 26.16 μg/mL) [32], and other amides [24] demonstrated the potential to interfere with critical physiological processes in mosquito larvae, particularly in *An. albimanus* Wiedemann, 1821, *An. gambiae* Giles, 1902, *An. quadrimaculatus* Say, 1824, *An. sinensis* Wiedemann, 1828, *An. subpictus* Grassi, 1899, *An. stephensi* Liston, 1901, *Ae. aegypti*, *Ae. albopictus* (Skuse, 1894), *Ae. togoi* Theobald, 1907, *Ae. subpictus* Grassi, 1899, *Culex quinquefasciatus* Say, 1823, *Cx. pipiens pallens* Coquillett, 1898, and *Cx. tritaeniorhynchus* Giles, 1901, contributing to the population reduction in these vectors without compromising local biodiversity.

The adoption of amides for mosquito control not only reduces the risks associated with synthetic chemicals but also promotes a more stable ecological balance by preserving natural predators and other species that play crucial roles in aquatic environments [33,34].

Regarding genus Piperaceae plants, *Piper purusanum* Yunck. is a shrub characterized by alternate ovoid leaves measuring 8.7 cm in length, with an oblique base and a petiole of about 0.3 cm, with erect inflorescence around 1.5 cm long, along with a sheath, midrib, secondary veins, stipules, and a stem and branches covered in spikes. It has a restricted distribution in the State of Amazonas, Brazil, as reported by Guimarães et al. [35]. The first report of the biological activities of the essential oil and its major compounds from this plant against *Ae. aegypti* and *An. darlingi* larvae, as well as their low toxicity to non-target aquatic animals, is described in detail in our study [13].

In this context, we hypothesize that piplartine could be an effective alternative for larval control, particularly against malaria and dengue vectors, with minimal toxic effects on non-target organisms, making it a safe and sustainable option for vector management. Thus, this study aimed to isolate and identify the amide piplartine from a crude leaf extract of *P. purusanum* using bioassay-guided methods. We investigated its larvicidal activity and mechanism of action against *Ae. aegypti* and *An. darlingi* to determine its effectiveness in vector control. Additionally, we evaluated its impact on non-target organisms to assess its environmental safety.

## 2. Results

### 2.1. Chemical Characterization of Piplartine

**Figure 1:**
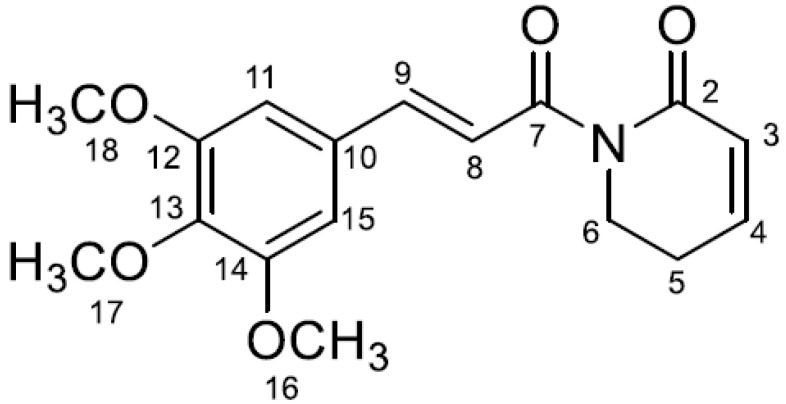
Structure of piplartine.

Compound **1** was isolated as white crystalline needles. Its MS/MS spectrum presented a base peak at *m/z* 221 (*m/z* 318 → 221), which is consistent with the structure of the amide alkaloid piplartine (Figure 1) [30,31,32]. Its ^1^H NMR spectrum displayed signals consistent with the expected structure (Supplementary Information), including olefinic protons at δ_H_ 7.61 (*d*, 15.9 Hz), 7.38 (*d*, 15.9 Hz), 7.08 (*dt*, 9.8; 4.3 Hz), and 6.01 (*dt*, 9.8; 1.8 Hz). Additionally, an aromatic proton was observed at δ_H_ 6.92 (*s*), methylene protons at δ_H_ 2.51 (*tdd*, 6.6; 4.3; 1.8 Hz) and 3.99 (*t*, 6.6 Hz), and methoxy groups at δ_H_ 3.80 (*s*) and 3.88 (*s*) (Table 1). Based on these data, compound **1** was confirmed as piplartine.

### 2.2. Larvicidal Activity Assay

**Figure 2:**
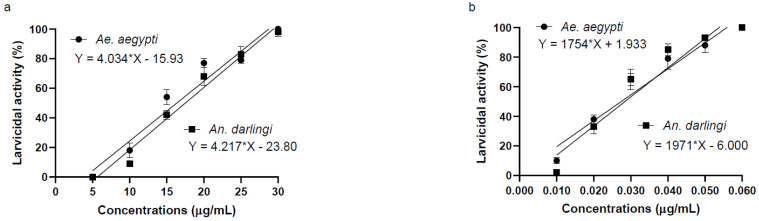
Mortality of *Ae. aegypti* and *An. darlingi* larvae following 48 h exposure to piplartine (**a**) and the positive control *α*-cypermethrin (**b**). No mortality of larvae was observed in the negative control DMSO at concentrations ranging from 5 to 30 μg/mL.

Piplartine demonstrated larvicidal activity against *Ae. aegypti* and *An. darlingi* larvae, with mortality rates ranging from 18.00 ± 5% to 100 ± 0% (R^2^ = 0.9442) for *Ae. aegypti* and from 9.00 ± 2% to 98.00 ± 5% (R^2^ = 0.9672) for *An. darlingi*. The LC_50_ was 14.56 μg/mL for *Ae. aegypti* and 26.44 μg/mL for *An. darlingi*. Additionally, the relative potency of piplartine was calculated as 0.0017 and 0.0013 for *Ae. aegypti* and *An. darlingi*, respectively. These findings, presented in Figure 2 and Table 2, confirm the effectiveness of piplartine as a larvicide. It is important to highlight that no mortality was observed in the negative control group, validating the experimental setup.

In contrast, α-cypermethrin showed significantly higher larvicidal potency. For *Ae. aegypti*, it caused mortality rates between 38.00 ± 3% and 100 ± 0% (R^2^ = 0.9251) with an LC_50_ of 0.023 μg/mL. For *An. darlingi*, mortality ranged from 33.00 ± 5% to 100 ± 0% (R^2^ = 0.9144) with an LC_50_ of 0.043 μg/mL. Statistical analysis revealed a highly significant difference in LC_50_ values among the treatments (F(3, 8) = 705.8, *p* < 0.0001).

These results highlight the potential of piplartine as a larvicide, although its potency is lower than that of α-cypermethrin. This distinction is critical for selecting appropriate compounds in vector control strategies.

### 2.3. Measurement of Reactive Oxygen and Nitrogen Species (RONS)

**Figure 3:**
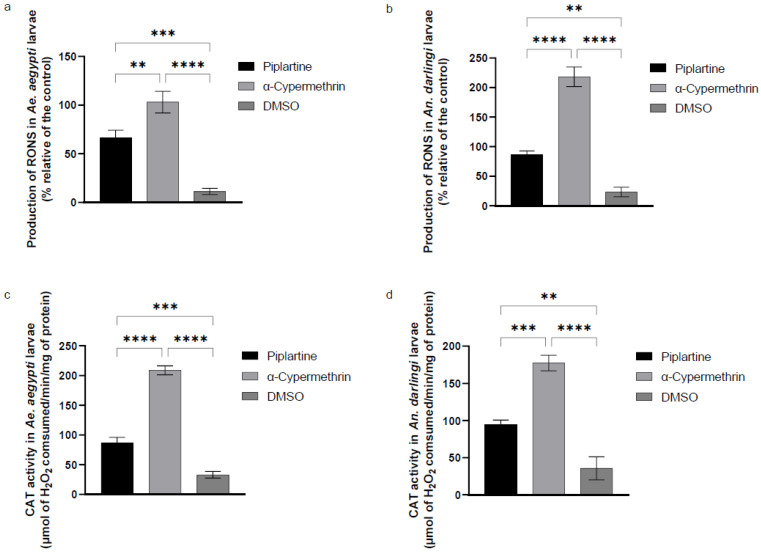
Production of reactive oxygen and nitrogen species (RONS) (**a**,**b**) and changes in catalase (CAT) activity (**c**,**d**) in *Ae. aegypti* and *An. darlingi* larvae after exposure to piplartine (30 μg/mL) compared to α-cypermethrin (0.060 μg/mL) and DMSO (30 μg/mL). For RONS, the statistical values for *Ae. aegypt*i were F(2, 6) = 100.6, *p* < 0.0001, while for *An. darlingi*, they were F(2, 6) = 234.5, *p* < 0.0001. Regarding CAT activity, the results for *Ae. aegypti* were F(2, 6) = 425.4, *p* < 0.0001, and for *An. darlingi*, F(2, 6) = 117.8, *p* < 0.0001. ** *p* = 0.0034, *** *p* = 0.0004, **** *p* < 0.0001.

Piplartine significantly increased the production of RONS in *Ae. aegypti* and *An. darlingi* larvae, with levels reaching 66.67 ± 7% and 86.33 ± 6% relative to the control, respectively. These RONS levels were considerably higher than those observed in the negative control group (DMSO), which induced substantially lower levels of 11.00 ± 3% in *Ae. aegypti* and 23.00 ± 8% in *An. darlingi*. In comparison, α-cypermethrin triggered the highest RONS production in both vector species, with levels reaching 103.00 ± 3% in *Ae. aegypti* and an even more pronounced 218.30 ± 1% in *An. darlingi*. Statistical analysis confirmed significant differences among treatments (F(2, 6) = 100.6, *p* < 0.0001 for *Ae. aegypti*; F(2, 6) = 234.5, *p* < 0.0001 for *An. darlingi*). These findings are illustrated in Figure 3a,b.

### 2.4. Catalase (CAT) Activity Assay

The results presented in Figure 3c,d indicate an increase in CAT activity in *Ae. aegypti* and *An. darlingi* larvae, with values of 87.00 ± 9 and 94.67 ± 9 μmol of H_2_O_2_ consumed per minute per mg of protein, respectively, compared to the DMSO control group, which exhibited significantly lower activity levels of 33.00 ± 5 and 35.15 ± 8 μmol of H_2_O_2_ consumed per minute per mg of protein, respectively. In contrast, α-cypermethrin demonstrated the highest CAT activity, with values of 209.00 ± 7 and 117.00 ± 1 μmol of H_2_O_2_ consumed per minute per mg of protein for both groups of mosquito larvae tested, respectively. Statistical analysis confirmed the significant differences among treatments (F(2, 6) = 425.4, *p* < 0.0001 for *Ae. aegypti*; F(2, 6) = 117.8, *p* < 0.0001 for *An. darlingi*).

These findings highlight the differential impact of piplartine and α-cypermethrin on CAT activity, suggesting a stronger oxidative stress response in larvae exposed to α-cypermethrin. This variability in enzymatic activity provides critical insights into the biochemical mechanisms associated with the larvicidal effects of these products.

### 2.5. Glutathione S-Transferase (GST) Activity Assay

**Figure 4:**
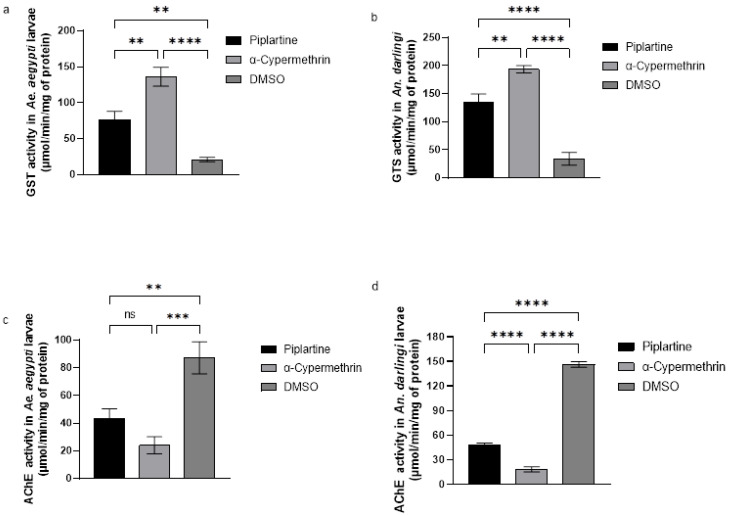
Changes in glutathione S-transferase (GST) (**a**,**b**) and acetylcholinesterase (AChE) activities (**c**,**d**) in Ae. aegypti and An. darlingi larvae following exposure to piplartine (30 μg/mL), compared with α-cypermethrin (0.060 μg/mL), and DMSO (30 μg/mL). For GST, the statistical values for Ae. aegypti were F(2, 6) = 91.20, *p* < 0.0001, while for An. darlingi, they were F(2, 6) = 147.9, *p* < 0.0001. Regarding AChE activity, the results for Ae. aegypti were F(2, 6) = 42.39, *p* < 0.0001, and for An. darlingi, F(2, 6) = 1409, *p* < 0.0001. ** *p* = 0.0010, *** *p* = 0.0003, **** *p* < 0.0001. ns—not significant.

A significant increase in GST activity was observed in *Ae. aegypti* and *An. darlingi* larvae following exposure to piplartine, with values reaching 76.00 ± 2 and 134.00 ± 5 μmol/min/mg protein, respectively. These levels were substantially higher than the negative control (DMSO), which showed GST activity of 20.00 ± 3 and 34.00 ± 1 μmol/min/mg protein, respectively. Conversely, α-cypermethrin induced the highest GST activity, with values of 136.00 ± 3 and 193.00 ± 6 μmol/min/mg protein for *Ae. aegypti* and *An. darlingi*, respectively, with statistical differences between treatments (F(2, 6) = 91.20, *p* < 0.0001 for *A. aegypti*; F(2, 6) = 147.9, *p* < 0.0001 for *An. darlingi*), as illustrated in Figure 4a,b.

These results highlight the distinct impact of piplartine and α-cypermethrin on GST activity, with α-cypermethrin inducing a more pronounced enzymatic response. This enhanced GST activity likely reflects the larvae’s oxidative stress response to these products and provides key insights into their biochemical modes of action.

### 2.6. Acetylcholinesterase (AChE) Activity Assay

A significant decrease in AChE activity was observed in *Ae. aegypti* and *An. darlingi* larvae after exposure to piplartine, with activity levels recorded at 43.33 ± 7 and 48.00 ± 2 μmol/min/mg of protein, respectively. This reduction indicates that piplartine effectively inhibits AChE activity in both species. Similarly, larvae exposed to α-cypermethrin showed even lower AChE activity, with values of 24.00 ± 6 and 18.33 ± 3 μmol/min/mg of protein for both mosquito larvae tested, respectively, confirming its strong inhibitory effect on AChE. On the other hand, no inhibition of AChE activity was observed in larvae treated with the negative control (DMSO), which exhibited significantly higher activity levels of 87.33 ± 1 and 146.30 ± 3 μmol/min/mg of protein for both mosquito larvae, respectively. These findings are represented in Figure 4c,d, which clearly illustrate the differences between treatments.

Statistical analyses further validated these observations, showing highly significant differences in AChE activity among the groups (F(2, 6) = 42.39, *p* < 0.0001 for *Ae. aegypti*; F(2, 6) = 1409, *p* < 0.0001 for *An. darlingi*). These results underscore the role of piplartine and α-cypermethrin as potent AChE inhibitors, highlighting their potential mechanisms of action and implications for mosquito larvicidal strategies.

### 2.7. Mixed-Function Oxidase (MFO) Activity Assay

**Figure 5:**
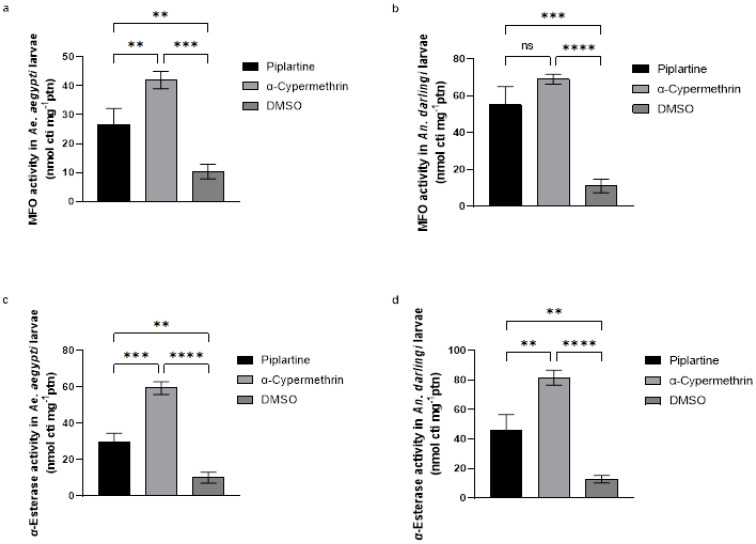
Alterations in mixed function oxidase (MFO) activity (**a**,**b**) and α-esterase activity (**c**,**d**) in *Ae. aegypti* and *An. darlingi* larvae following exposure to piplartine (30 μg/mL), compared to α-cypermethrin (0.060 μg/mL) and DMSO (30 μg/mL). For MFO, the statistical values for *Ae. aegypti* were F(2, 6) = 49.42, *p* < 0.0001, while for *An. darlingi*, they were F(2, 6) = 68.70, *p* < 0.0001. While for α-esterase activity, the results for *Ae. aegypti* were F(2, 6) = 116.7, *p* < 0.0001, and for *An. darlingi*, F(2, 6) = 78.78, *p* < 0.0001. ** *p* = 0.0052, *** *p* = 0.0001, **** *p* < 0.0001, ns—not significant.

The results in Figure 5a,b reveal significant alterations in MFO activity in *Ae. aegypti* and *An. darlingi* larvae after exposure to piplartine. MFO activity increased to 26.67 ± 5 nmol cti mg⁻¹ protein in *A. aegypti* and 55.00 ± 1 nmol cti mg⁻¹ protein in *An. darlingi*, indicating a substantial enzymatic response to the compound. Similarly, exposure to α-cypermethrin induced even greater MFO activity, with recorded values of 42.50 ± 3 nmol cti mg⁻¹ protein in *Ae. aegypti* and 69.70 ± 2 nmol cti mg⁻¹ protein in *An. darlingi*. These results underscore the pronounced effect of both products in enhancing MFO activity in larvae.

Differently, larvae treated with the negative control (DMSO) exhibited only a slight increase in MFO activity, with values of 10.33 ± 3 nmol cti mg⁻¹ protein, demonstrating the limited impact of DMSO on this enzyme system. Statistical analyses confirmed highly significant differences in MFO activity among the treatment groups (F(2, 6) = 49.42, *p* < 0.0001 for *Ae. aegypti*; F(2, 6) = 68.70, *p* < 0.0001 for *An. darlingi*). This evidence highlights the ability of both piplartine and α-cypermethrin to stimulate MFO activity. This response suggests a possible role of MFO in the detoxification process or as a target for larvicidal activity in these mosquito species.

### 2.8. α- and β-Esterase Activity Assay

Piplartine induced notable increases in α-esterase activity in *Ae. aegypti* and *An. darlingi* larvae, with activity levels reaching 29.33 ± 5 and 46.33 ± 1 nmol cti mg⁻¹ protein, respectively. These values were significantly higher compared to the negative control (DMSO), which caused only modest increases of 10.20 ± 3 and 12.67 ± 2 nmol cti mg⁻¹ protein, respectively. Inversely, exposure to α-cypermethrin resulted in the highest levels of α-esterase activity, with values of 59.33 ± 3 and 81.67 ± 5 nmol cti mg⁻¹ protein for both mosquito larvae, respectively. Statistical analyses confirmed these differences as significant (F(2, 6) = 116.7, *p* < 0.0001 for *Ae. aegypti*; F(2, 6) = 78.78, *p* < 0.0001 for *An. darlingi*), as depicted in Figure 5c,d.

**Figure 6:**
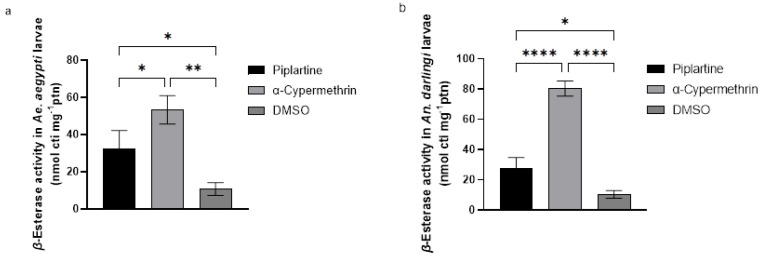
Alterations in β-esterase activity (**a**,**b**) in *Ae. aegypti* and *An. darlingi* larvae following exposure to piplartine (30 μg/mL), compared to α-cypermethrin (0.060 μg/mL) and DMSO (30 μg/mL). For β-esterase activity, the statistical values for *Ae. aegypti* were F(2, 6) = 23.59, *p* < 0.0001, while for *An. darlingi*, they were F(2, 6) = 147.7, *p* < 0.0001. * *p* = 0.0032, ** *p* = 0.0011, **** *p* < 0.0001.

Similar findings were observed for β-esterase activity. Exposure to piplartine increased β-esterase levels to 32.00 ± 1 and 27.67 ± 7 nmol cti mg⁻¹ protein in both mosquito larvae tested, respectively. These increases were more significant than those induced by DMSO, which resulted in β-esterase activity of 10.67 ± 3 and 10.33 ± 2 nmol cti mg⁻¹ protein for the respective species. However, α-cypermethrin again triggered the highest β-esterase activity, with values of 53.33 ± 7 and 80.33 ± 5 nmol cti mg⁻¹ protein for both larvae, respectively. Statistical analyses confirmed significant differences among the treatments (F(2, 6) = 23.59, *p* < 0.0001 for *Ae. aegypti*; F(2, 6) = 147.7, *p* < 0.0001 for *An. darlingi*), as illustrated in Figure 6a,b.

These outcomes indicate that both piplartine and α-cypermethrin influence esterase activity, with α-cypermethrin exerting a more pronounced effect. The differential responses highlight the potential of these enzymes as biomarkers for larvicidal activity and provide insights into the biochemical pathways targeted by these products.

### 2.9. Potential Lethal Effects on Entomological Fauna Assay

The Kaplan–Meier and log-rank (Mantel–Cox) analyses revealed significant differences in the survival curves of non-target aquatic insects, including Notonectidae, Gerridae, Corixidae, Nepidae, Mesoveliidae, Belostomatidae, Chironomidae, Neocoridae, Caenidae, and Hydrophilidae, over a 30-day period. Exposure to piplartine and DMSO, both at a concentration of 264.4 μg/mL, resulted in 100% survival, demonstrating the absence of toxicity in these treatments.

**Figure 7:**
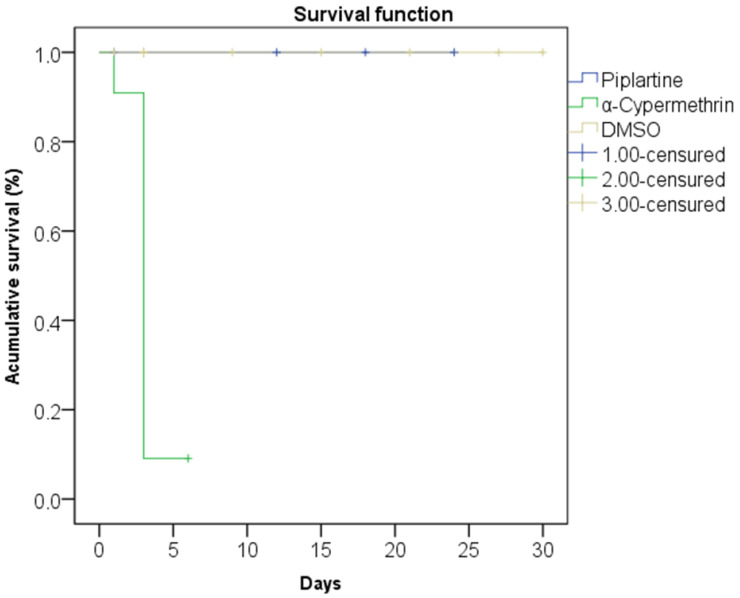
The Kaplan-Meier method, followed by the Log-rank (Mantel-Cox) test, were used to show survival curves of non-target animals over a 30-day period after exposure to piplartine (264.4 μg/mL), α-cypermethrin (0.025 μg/mL), and DMSO (264.4 μg/mL). The results indicated a significant difference with Chi-square = 32.657, degrees of freedom = 2, and *p* < 0.0001.

In contrast, exposure to α-cypermethrin at 0.025 μg/mL, a concentration 10.5 times lower than that of piplartine and DMSO, demonstrated rapid and acute toxicity, resulting in a survival rate of only 9.1% within just three days. This marked disparity underscores the safety of piplartine and DMSO compared to the pronounced toxicity of α-cypermethrin. Statistical analyses confirmed these findings, with a chi-squared value of 32.657, degrees of freedom = 2, and a highly significant *p*-value < 0.0001. Figure 7 illustrates the distinct survival curves for each treatment group.

Further evidence from Cox regression analysis (Table 3) supports the conclusion that α-cypermethrin poses a significantly higher mortality risk for non-target species. With an odds ratio of 54.598 and a *p*-value of 0.030, animals exposed to α-cypermethrin were 54.6 times more likely to die than those treated with piplartine or DMSO. Conversely, both piplartine and DMSO, at 264.4 μg/mL, had odds ratios near 1.0 and *p*-values of 1.000, indicating no impact on mortality.

The covariate analysis showed that the order of insects did not significantly influence mortality outcomes, with an odds ratio of 1.005 and a *p*-value of 0.964. These results emphasize the significant toxicity of α-cypermethrin compared to the relative safety of piplartine and DMSO for the tested non-target organisms (Table 3). Such findings are critical for environmental risk assessments and the development of safer alternatives for pest management.

## 3. Discussion

The Piperaceae family is a diverse group of plants consisting of five genera and over 3000 species, characterized by their versatile stem structures and pinnate leaves [37]. These species are predominantly distributed across tropical and subtropical regions [38]. Among them, the genus *Piper*, comprising more than 1000 species, is particularly notable for its abundance of secondary metabolites, including amides and alkaloids, which exhibit a wide range of biological activities with a pronounced effect on arthropods [39].

Among amides, piplartine, also known as piperlongumine, is a bioactive compound that was first isolated in 1967 from the petroleum ether extract of the dried stem bark and roots of *P. longum* Linnaeus, yielding 2.5 g obtained using classical techniques for isolation, purification, and characterization including column chromatography, ultraviolet (UV) spectroscopy, nuclear magnetic resonance (NMR) spectroscopy, and mass spectrometry, which collectively confirmed the compound structure and identity [40]. Later, the compound was also described in *P. tuberculatum Jacq. [41] and P. aborescens* Roxb. [42].

To the best of our knowledge, this is the first report describing the presence of piplartine in the leaves of *P. purusanum*, in which the fragmentation patterns and chemical data were consistent with those previously reported for piplartine isolated from the roots, fruits, and leaves and seed of *P. aborescens* Roxb., *P. cernuum* Vell, *P. longum* L., and *P. tuberculatum* Jacq. [41,42,43,44,45,46].

Regarding its chemical characteristics, piplartine consists of two carbonyl groups. The first is directly attached to carbon number 7, which is connected to the pyridine ring, while the second is located on carbon number 2 of the pyridine ring. Additionally, there are three methoxy groups attached to carbons 12, 13, and 14 (Figure 1), interacting significantly for the biological activity of the molecule [46].

The methoxy groups, being electron-donating, increase the electronic density in the aromatic ring, influencing its polarity and stability, which promotes solubility and interaction with biomolecules [38]. On the other hand, the carbonyl groups, being electrophilic, participate in important interactions with proteins and enzymes, such as hydrogen bonding, and contribute to the molecule reactivity and affinity with biological targets [44].

This interaction between the methoxy and carbonyl groups creates a balance that enhances solubility, permeability, and biological activity, which is essential for the insecticidal properties, as described with *Ae. aegypti* larvae; with a decrease in L3, the adult development time from 3 days to 1 day was observed at concentrations of 1 and 10 μg/mL of piplartine, respectively, compared to the negative control, which ranged from 4 to 5 days [31]. Additionally, larvicidal activity was noted, with an LC_50_ of 155.5 μg/mL [30]. This larvicidal activity was also reported against *Ae. aegypti* and *An. darlingi* larvae, described in Table 2.

According to Maleck et al. [30], several significant changes were observed in the digestive tract, indicating cellular stress followed by apoptosis on *Ae. aegypti* larvae. These changes include intense cytoplasmic vacuolization, shortened and sparse microvilli, the presence of myelin, and disorganized cellular structures. Additionally, some nuclei in the midgut cell exhibited DNA extraction, further highlighting the extent of cellular damage. In fact, the observation of myelin in mosquito larvae cells could indicate cellular stress, as this structure is often associated with cell damage and breakdown in other biological contexts [47].

The cellular damage described by Maleck et al. [30] suggests that piplartine induces oxidative stress in *Ae. aegypti* larvae, even though the authors did not perform biochemical or enzymatic assays to investigate this effect directly. In contrast, our findings, as shown in Figure 3a,b, provide clear evidence that piplartine at a concentration of 25 μg/mL significantly promotes the overproduction of RONS in both *Ae. aegypti* and *An. darlingi* larvae, therefore elucidatingClick or tap here to enter text. a larvicidal mechanism of action. Indeed, a recent review conducted by da Azevedo Da Silva et al. [43] confirmed the potential of this compound in induced RONS and apoptosis cells.

Exposure to bioactive compounds, such as piplartine, causes significant cellular damage in mosquito larvae, disrupting vital functions and leading to death [48]. The main alterations observed include disorganization of the intestinal epithelium, characterized by intense cytoplasmic vacuolization, reduction or destruction of microvilli, and the general disruption of epithelial cell structures, which are often attributed to the induction of oxidative stress, evidenced by the excessive production RONS, which promote lipid peroxidation and compromise the integrity of cellular membranes [49]. Furthermore, nuclear alterations, such as the extraction of genetic material, suggest the activation of programmed cell death processes (apoptosis) or necrosis [47].

The overproduction of RONS contributes to an increased generation of free radicals, which can cause significant damage to critical biomolecules such as lipids, proteins, carbohydrates, and nucleic acids [48]. This process disrupts the organism antioxidant defense system, creating an imbalance that exacerbates oxidative stress [50]. Due to their reactivity, these species can interact with a wide range of biomolecules, amplifying the complexity and extent of oxidative damage within the organism [51]. These findings highlight the potential of bioactive compounds such as piplartine as larvicidal agents for vector control, acting through multiple cellular mechanisms [52].

The correlation between larvicidal or insecticidal activity and the production of RONS has been clearly demonstrated in various arthropod species, including *Ae. aegypti*, *An. darlingi*, *Cx. quinquefasciatus* Say, 1823, *An. stephensi* Liston, 1901 (Culicidae), *Drosophila melanogaster* Meigen, 1830 (Drosophilidae), *Ephestia kuehniella* Zeller, 1879 (Pyralidae), and others. This effect was observed following exposure to essential oils and compounds such as β-caryophyllene, fenchone, 4-vinylcyclohexene 1,2-monoepoxide, and 4-vinylcyclohexene diepoxide, which are derived from plants like *P. tuberculatum* Jacq., *P. purusanum* C.DC., *P. alatipetiolatum* Yunck. (Piperaceae), *Tetradenia riparia* (Hochstetter) Codd (Lamiaceae), and *Cymbopogon citratus* (DC.) Stapf. (Poaceae), as well as synthetic insecticides such as α-cypermethrin, temephos, and imidacloprid, which have also been linked to similar effects [13,48,50,51,52,53].

The RONS in mosquito larvae, induced by amides such as piplartine, triggers significant oxidative stress that causes critical alterations in the organism antioxidant and metabolic systems [54]. This increase in RONS acts as a signal for the activation of antioxidant defense mechanisms, leading to an elevation in the activity of protective enzymes such as catalase, GST, MFO, and esterases (Figure 3, Figure 4, Figure 5 and Figure 6). However, AChE activity often decreases, suggesting a direct impact of the amides on essential neurological and metabolic processes [53] (Figure 4c,d).

CAT plays a pivotal role in neutralizing hydrogen peroxide, converting it into water and oxygen [7]. Elevated CAT activity indicates the organism effort to mitigate the deleterious effects of oxidative stress [49]. Complementarily, GST acts by conjugating reduced glutathione to reactive species, facilitating the detoxification of xenobiotic compounds and lipid peroxidation products [47]. MFOs are essential for the oxidative metabolism of xenobiotics, while esterases contribute to the degradation of esters and other toxic compounds [55].

On the other hand, the decline in AChE activity can be attributed to two primary factors. Firstly, the accumulation of RONS and associated oxidative damage may compromise the structure and functionality of the enzyme, reducing its efficiency [56]. Secondly, amides such as piplartine may directly inhibit AChE by interacting with its active sites, an effect observed in several studies on insecticidal compounds [57]. This reduction in AChE activity has significant implications, as the enzyme is fundamental for regulating neurotransmission [58]. Its inhibition can lead to acetylcholine accumulation in synapses, resulting in neuromuscular dysfunction, paralysis, and eventually larval death [53].

The decline in AChE activity, in contrast to the increased activity of other defense enzymes, highlights the complexity of the organism response to induced oxidative stress [14]. While antioxidant and metabolizing enzymes attempt to mitigate cellular damage, AChE inhibition compromises neurological function, intensifying the toxic effects of amides [54]. This imbalance between defense mechanisms and oxidative damage represents one of the primary larvicidal mechanisms of amides, emphasizing their effectiveness in vector control [59,60,61].

Regarding the comparative toxicity assessment of piplartine and α-cypermethrin for non-target aquatic insects, the results obtained in this study provide robust and conclusive evidence regarding the toxicological differences between piplartine and α-cypermethrin, two compounds with potential for vector control but with markedly distinct toxicity profiles. Piplartine, even at a high concentration of 264.4 μg/mL, showed no observable toxicity, resulting in 100% survival for Notonectidae, Gerridae, Corixidae, Nepidae, Mesoveliidae, Belostomatidae, Chironomidae, Neocoridae, Caenidae, and Hydrophilidae over a 30-day period. DMSO, used as a solvent control, also demonstrated equivalent safety, causing no negative impacts on the tested species. In contrast, α-cypermethrin, applied at a substantially lower concentration (0.025 μg/mL), induced acute toxicity in all species, with a survival rate of only 9.1% after three days of exposure.

In fact, for example, α-cypermethrin and several synthetic larvicides, including lambda-cyhalothrin, thiamethoxam, and temephos, have been shown to be highly toxic to non-target species such as *Cyprinus* sp. (Cyprinidae), *Diplonychus* sp. (Heteroptera), *Toxorhynchites* sp. (Culicidae), *Anisops* sp. (Hemiptera), and *Gambusia* sp. (Poeciliidae), with LC_50_ values ranging from 0.22 to 5.82 μg/mL [9,13,14]. In a recent study, the acute and rapid toxicity of α-cypermethrin at a concentration of 0.39 μg/mL was observed on *T. haemorrhoidalis*, *A. bouvieri*, and *D. indicus*, resulting in the complete mortality (100% death rate) of these animals [50].

These results highlight the importance of selecting fewer toxic alternatives for vector control, considering the environmental impacts that insecticides can have on non-target organisms, especially those inhabiting aquatic environments [13]. While α-cypermethrin is widely used for its effectiveness, its devastating effects on non-target species, as evidenced by Cox regression and Kaplan–Meier analysis, emphasize the need for more sustainable strategies [62]. The Cox regression revealed that the mortality associated with α-cypermethrin was 54.6 times higher than that observed with piplartine or DMSO, indicating pronounced lethality that could compromise aquatic biodiversity [63].

On the other hand, piplartine demonstrated a favorable safety profile, likely due to its selective action on specific physiological mechanisms in mosquitoes without affecting other groups of aquatic insects [64]. The absence of mortality suggests a wide safety margin and positive ecological compatibility of piplartine with non-target aquatic organisms [65]. This aspect is particularly relevant when considering these species’ diversity and ecological importance in aquatic environments, as they play essential roles in maintaining aquatic ecosystems, such as predators, decomposers, and prey for other animals [15].

Moreover, the covariate analysis revealed that the insect order did not influence the mortality outcomes, reinforcing the consistency of the results and suggesting that the observed response was solely due to the treatments rather than variability among species [66]. The statistical robustness of the results, supported by highly significant *p*-values, further strengthens the confidence in the conclusions that piplartine poses a low risk to non-target aquatic organisms in contrast to α-cypermethrin [67].

It is important to highlight that many of the non-target aquatic insects tested in this study inhabit aquatic environments that often face varying degrees of pollution, which may have influenced their ability to cope with environmental stressors [12,55]. In contaminated aquatic ecosystems, many of these organisms have developed defense systems adapted to detoxifying xenobiotics, such as antioxidant enzymes and phase I and II metabolizing enzymes [12]. Such adaptations may have contributed to the resistance observed in several of the tested species, as these insects may be more efficient in neutralizing the toxic effects of compounds present in the environment, including pesticides [68]. This factor may partially explain the absence of mortality observed in the groups treated with piplartine and DMSO, as the ability to metabolize and excrete toxic substances may have acted as a protective mechanism against the compound effects [69].

In contrast, α-cypermethrin, being a broad-spectrum insecticide with a less selective mode of action, likely interferes directly with critical physiological pathways, causing irreparable damage to non-adapted aquatic insects [12]. The observed acute toxicity, even at low concentrations, can be attributed to the inability of these species to properly metabolize the compound, leading to the accumulation of free radicals and irreversible cellular damage [70].

The presented results have crucial implications for environmental risk assessment and the development of safer alternatives for mosquito control, such as Culicidae [71]. Piplartine, with its much lower toxicity to non-target aquatic insects, positions itself as a promising alternative in vector control programs while also preserving aquatic biodiversity, and its use could significantly reduce the adverse ecological impacts caused by traditional insecticides, such as α-cypermethrin, which pose high risks to non-target organisms [68].

## 4. Material and Methods

### 4.1. Chemicals and Reagents

3,3′,5,5′-Tetramethylbenzidine dihydrochloride hydrate (TMBZ) (98%), phosphate buffer (pH 7.3), acetonitrile (99%), para-nitrophenyl acetate (98%), potassium phosphate buffer (pH 7.4), sodium phosphate buffer (pH 7.2), *α*-naphthyl acetate (98%), *β*-naphthyl acetate (99%), sodium acetate (NaOAc) (99%), xanthine oxidase (lyophilized powder, 0.4–1 units/mg), bovine serum albumin (pH 7, 98%) (BSA), hydrogen peroxide (H_2_O_2_) (30%), 1-chloro-2,4-dinitrobenzene (CDNB) (99%), Bio-Rad reagent, ethylenediaminetetraacetic acid (EDTA) (98%), glutathione (GSH) (analytical standard), 5,5′-dithiobis(2-nitrobenzoic acid) (DTNB) (99%), acetylthiocholine iodide (AChI) (99%), 2′,7′-dichlorodihydrofluorescein diacetate (DCFH-DA) (95%), 2′,7′-dichlorofluorescein (DCF) (90%), dimethyl sulfoxide (DMSO) (99%), *α*-cypermethrin (PESTANAL^®^, analytical standard), solvents HPLC-grade, as well as analytical-grade, including hexane (Hex), ethyl acetate (EtOAc), dimethyl sulfoxide (DMSO), chloroform (CHCl_3_), and methanol (MeOH) were purchased from Merck and Tedia, while nitro blue tetrazolium chloride (NBT) (98%) was purchased from Thermo Fisher Scientific Inc. Ultrahigh-purity water was obtained by Milli Q system (Millipore, Bedford, MA, USA).

### 4.2. General Experimental Procedures

The ^1^H NMR experiment was acquired in deuterated methanol (CD_3_OD) on a Bruker AVANCE III HD spectrometer operating at 11.75 Tesla (500.13 MHz). All ^1^H NMR chemical shifts (*δ*) were presented in parts per million (ppm) relative to the tetramethylsilane (TMS) signal at 0.00 ppm, and the coupling constants (*J*) were given in Hertz. For mass spectrometry analysis, the sample was resuspended in methanol (HPLC-grade), creating stock solutions (1 mg/mL). An aliquot (5 μL) of the stock solutions was further diluted to 5 μg/mL and analyzed by direct infusion into an ion trap mass spectrometer, model LCQ Fleets (Thermo Scientific, San Jose, CA, USA) equipped with atmospheric pressure chemical ionization (APCI) sources in positive mode. Semi-preparative HPLC analysis was performed on a Shimadzu UFLC system equipped with a Luna C18(2) column (250 mm × 10 mm, 5 μm). Column chromatography (CC) was performed on silica gel (70–230 mesh particle size) purchased from Merck, Brazil.

### 4.3. Obtaining the Methanolic Extract

All procedures for the collection and identification of *P. purusanum* species were detailed in our previous study [14]. The methanolic extract of *P. purusanum* (MEPp) was obtained using 200 g of powdered leaves macerated in 4 L of 100% MeOH (analytical grade) at room temperature (30 ± 5 °C) for seven days. After filtration using Whatman (no. 43) filter paper (Sigma-Aldrich, São Paulo, Brazil), the extract was concentrated under reduced pressure (72 mbar) using a rotary evaporator (Fisatom, model 558, São Paulo, Brazil) at 40 °C until all the solvent was removed. Then, the residual solvent was eliminated in an oven at 40 °C for 72 h. The resulting MEPp crude weighed 71.974 g (yielding 35.98%) and was stored in an amber glass container at 4 °C [72].

### 4.4. Partitioning of the Methanolic Extract

The MEPp (20 g) was sequentially partitioned into Hex (100%) (2.8 g), EtOAc (100%) (6.8 g), CHCl_3_ (100%) (4.7 g), and water (5.7 g). These fractions were concentrated using a rotary evaporator at 40 °C. The water portion was freeze-dried (Alpha 1–2 LDplus, Martin Christ, Germany) following the methodology previously reported [73]. Subsequently, a concentration of 100 μg/mL for each fraction previously prepared in 1 mL of DMSO was investigated for potential larvicidal activity against *A. aegypti* and *An. darlingi* [74]. The CHCl_3_ fraction, which caused 100% larval mortality for both vectors, was chromatographed on a silica gel column (Merck 70–230 mesh, 300 g, 4.5 i.d × 60 cm) and successively eluted with Hex (100%) (0.5 g), Hex/EtOAc (8:2) (2 g), EtOAc (100%) (1.8 g), and MeOH (100%) (0.4). Then, each fraction (100 μg/mL) was reanalyzed for larvicidal activity, leading the identification of the Hex/EtOAc fraction, which caused 91.5 and 97.2% mortality in *A. aegypti* and *An. darlingi* larvae, respectively.

### 4.5. HPLC Semi-Preparative Isolation

The Hex/EtOAc fraction (120 mg) was subjected to a separation by HPLC semi-preparative. The isocratic elution was performed from 0 to 30 min with 50% B (*v*/*v*) at a flow rate of 4.5 mL/min. A C18(2) column (250 mm × 10 mm, 5 μm) from Phenomenex (Torrance, CA, United States) was used for the fractionation, with monitoring by UV channels at 220 and 280 nm. Five injections of 30 mg each were loaded into the column using 100 μL of DMSO as the solvent, resulting in the isolation of compound **1** (97.8 mg).

### 4.6. Mosquito Rearing

The *Ae. aegypti* larvae were provided by the Malaria and Dengue Laboratory of the National Institute of Amazonian Research under controlled conditions of temperature of 26 ± 3 °C, relative humidity of 85%, and a 12 h light/12 h dark photoperiod, following the detailed methodology described [75]. Briefly, after hatching, the larvae were fed fish food until they reached the third instar, which were used for larvicidal bioassays.

*An. darlingi* larvae were collected using an entomological shell at Ramal Brasileirinho (latitude 3° 0′ 42.9″ S, longitude 59° 52′ 29.9″ W) in Manaus, Amazonas, Brazil, following the guidelines of the World Health Organization (WHO) Manual on Practical Entomology in Malaria [76]. After a 24 h acclimation period, the larvae were identified using a taxonomic key [77] and reared under the same conditions described above.

### 4.7. Larvicidal Activity Assay

The larvicidal assay was performed under controlled conditions of temperature of 26 ± 3 °C and relative humidity of 85%, following the Guidelines for Laboratory and Field Testing of Mosquito Larvicides [74]. In brief, third instar larvae of *Ae. aegypti* (*n* = 2.500) and *An. darlingi* (*n* = 1.250) were transferred for twenty-five recipients (200 mL) containing 99 mL of distilled water and concentrations ranging from 5 to 25 μg/mL of compound **1**, obtained from a stock solution of 25.5 mg/mL prepared in 1mL of DMSO. As the negative control, DMSO also was assessed in these concentrations, while *α*-cypermethrin was used as a positive control at concentrations ranging from 0.10 to 0.60 μg/mL.

The assay was performed in quintuplicate with five repetitions. The percentage of activity at each concentration was calculated after a 48 h exposure period using the following equation: Larvicidal activity (%) = number of dead larvae/number of larvae used × 100. The relative potency (RP) compound **1** was identified using the following equation: RP = LC_50_ of standard larvicidal/LC_50_ of natural product [36].

### 4.8. Homogenate Preparation

Homogenate preparation was performed at the end of the 48 h exposure period in the larvicidal assay. Fifty larvae each of *Ae. aegypti* and *An. darlingi*, treated with 25 μg/mL of compound **1**, DMSO, and 0.60 μg/mL of *α*-cypermethrin, were transferred to eight tubes (5 mL) homogenized with 2.5 mL of potassium phosphate buffer (pH 7.3 at 0.1 M), utilizing a tube vortex mixer for 5 min, followed by centrifugation at 4000 rpm for 5 min. The resulting supernatant was transferred to Eppendorf tubes (3 mL) stored at −4 °C for further biochemical analysis. Absorbance levels were measured using an LMR-96-8 microplate reader (Loccus^®^, Brazil) at the wavelength specific to each enzyme. The mean absorbance was then calculated based on three replicates [78].

### 4.9. Measurement of Reactive Oxygen and Nitrogen Species (RONS)

The measurement of RONS overproduction in *Ae. aegypti* and *An. darlingi* larvae was conducted according to the methodology reported [79] and adapted for larvae supernatant [48]. In brief, an aliquot of 5 μL of each supernatant (1:10 dilution), 5 μL of DCFH-DA, 40 μL of distilled water, and 150 μL of potassium buffer (pH 7.4 at 0.1 M) were transferred to a 96-well microplate, followed by incubation at 37 °C for 60 min. A standard curve was conducted using increasing concentrations of DCF, incubated in parallel, obtaining in a final concentration of 5 μL. The fluorescence resulting from DCFH oxidation was measured over a 10 min period in 30 s intervals using a SpectraMax plate reader (Molecular Devices, USA) with excitation at 488 nm and emission at 525 nm. Control groups included *α*-cypermethrin and DMSO. The rate of DCF formation was expressed as a percentage relative to the control groups. All assays were performed in triplicate for each supernatant.

### 4.10. Catalase (CAT) Activity Assay

CAT activity was determined using the methodology originally developed by Aebi [80] and later adapted by Abolaji et al. [48]. Briefly, 20 μL of each supernatant was mixed with a solution containing 180 μL of H_2_O_2_ (300 mM) and 1.8 mL of phosphate buffer (pH 7.3, 0.1 M) at 25 °C. The reduction of H_2_O_2_ was then monitored over a 2 min period in 10 s intervals, with absorbance measured at 240 nm using a UV–visible spectrophotometer. The results were expressed as μmol of H_2_O_2_ consumed per minute per mg of protein.

### 4.11. Glutathione S-Transferase (GST) Activity Assay

GST activity was measured using 1-chloro-2,4-dinitrobenzene (CDNB) as the substrate, following the method described by Habig and Jakoby [81]. Briefly, 20 μL of each supernatant was mixed with 270 μL of Solution A, which consisted of 2.5 mM EDTA, 10.5 μL of distilled water, 20 μL of 0.25 M phosphate buffer, and 500 μL of 0.1 M GSH (pH 7.0). The mixture was maintained at 25 °C, and then 10 μL of 25 mM CDNB was added. The absorbance was read at 340 nm in 10 s intervals over a period of 5 min. Finally, GST activity was expressed in μmol/min/mg protein. All experiments were carried out in triplicate.

### 4.12. Acetylcholinesterase (AChE) Activity Assay

The AChE activity assay was performed using a method initially described by Ellman et al. [82] and later adapted for a 96-well microplate by Abolaji et al. [48]. In a 96-well microplate, 135 μL of distilled water was mixed with 20 μL of DTNB (10 mM), 20 μL of phosphate buffer (pH 7.3, 100 mM), 20 μL of AChI (8 mM), and 20 μL of each supernatant. Absorbance readings were taken at a wavelength of 412 nm every 30 s over a 2 min period. AChE activity was expressed as μmol/min/mg protein. All procedures were conducted at 25 °C without light, and all experiments were performed in triplicate.

### 4.13. Mixed-Function Oxidase (MFO) Activity Assay

The MFO assay was performed following the protocol detailed in the Quantification Methodology for Enzyme Activity Related to Insecticide Resistance in *Aedes aegypti* from Vale et al. [83]. Before the assay, a sodium acetate (NaOAc) buffer was prepared by mixing 41.6 mL of 3 M sodium acetate with 450 mL of water and adjusting the pH to 5. The TMBZ solution was then prepared by dissolving 10 mg of TMBZ in 5 mL of methanol and 15 mL of 0.25 M NaOAc buffer (pH 5). For the assay, 200 μL of the TMBZ solution, 25 μL of 3% hydrogen peroxide, and 20 μL of each supernatant were added in each well of a microplate. The microplate was then incubated at room temperature for 10 min and subsequently read at 620 nm. The results were expressed as the mean number of cytochrome nanomoles per total mosquito protein with standard error (SE).

### 4.14. α- and β-Esterase Activity Assay

To each well, 200 μL of α-naphthyl acetate and β-naphthyl acetate (0.3 mM) in phosphate buffer were added to 10 μL of each supernatant. The mixture was incubated at room temperature for 15 min. Subsequently, 50 μL of Fast Blue reagent (30 mg in 3 mL Milli-Q water and 7 mL of 3% SDS) was added to each well. After an additional 5 min incubation, the production of *α*-naphthol and β-naphthol was measured at 570 nm. The results were expressed as mean micromoles (μmol) of *α*-naphthol and *β*-naphthol produced per mg of protein per minute ± standard error (SE), respectively [8].

### 4.15. Measurement of Total Protein

Total protein concentrations were measured by adding 300 μL of Bio-Rad reagent (diluted 1:5) to 10 μL of each supernatant, resulting in a final volume of 310 μL per well. Absorbance was then measured at a wavelength of 620 nm [8].

### 4.16. Potential Lethal Effects on Entomological Fauna Assay

The potential lethal effects of compound **1** on non-target aquatic animals was conducted following the methodology described by Sivagnaname and Kalyanasundaram [84] under controlled conditions of temperature (28 ± 2 °C) and relative humidity (80 ± 5%). The animals, including Hemiptera: Notonectidae (*n* = 152), Gerridae (*n* = 125), Corixidae (*n* = 204), Nepidae (*n* = 191), Mesoveliidae (*n* = 189), Belostomatidae (*n* = 271), and Neocoridae (*n* = 233); Ephemeroptera: Caenidae (*n* = 111); Coleoptera: Hydrophilidae (*n* = 119); and Diptera: Chironomidae (*n* = 127 larvae), were collected in the same habitat as *An. darlingi* larvae, with an entomological shell and identified using a taxonomic key [15].

The animals were separated into their respective orders and acclimated for 24 h in recipients (90 cm in diameter and 40 cm deep) containing 5 L of water from their breeding habitat. Then, they were transferred to recipients (500 mL) containing 400 mL of water from their breeding habitat and 264.4 μg/mL of compound 1 obtained by multiplying LC_90_ of 26.44 μg/mL (Table 1) by 10 [50]. DMSO was also used in this concentration, while α-cypermethrin was used at concentration of 0.025 μg/mL (Table 1).

Each group of organisms was divided equally among the three treatments for analysis, with Notonectidae (*n* = 51 per treatment), Gerridae (*n* = 42 per treatment), Corixidae (*n* = 68 per treatment), Nepidae (*n* = 64 per treatment), Mesoveliidae (*n* = 63 per treatment), Belostomatidae (*n* = 90 per treatment), Neocoridae (*n* = 78 per treatment), Caenidae (*n* = 37 per treatment), Hydrophilidae (*n* = 40 per treatment), and Chironomidae larvae (*n* = 42 per treatment). The assay was monitored over a 30-day exposure period to estimate the survival curve.

### 4.17. Statistical Analysis

The larvicidal activity percentages were analyzed using Probit analysis in IBM^®^ SPSS^®^ Statistics to estimate the LC_50_ and LC_90_ values, along with linear regression, chi-squared tests, and degrees of freedom calculations. The estimated LC_50_ and LC_90_ values, along with enzyme activity data, were then subjected to a one-way analysis of variance (ANOVA) followed by Tukey’s post hoc test (*p* < 0.05) to identify statistical differences among the treated groups using GraphPad Prism^®^ 9 software [85].

To analyze survival data, the Kaplan–Meier method was employed to construct survival curves, while the log-rank (Mantel–Cox) test was used to compare survival curves. Additionally, Cox regression analysis was performed to assess the influence of predictive factors (family and treatment). These survival analyses were conducted in IBM^®^ SPSS^®^ Statistics [86,87].

## 5. Conclusions

This study underscores the great potential of piplartine as an ecological and efficient alternative to conventional insecticides, such as α-cypermethrin, especially for mosquito larval control, such as *A. aegypti* and *An. darlingi*, without causing significant harm to non-target species. The absence of toxicity in non-target aquatic insects suggests that piplartine could be integrated into vector control strategies with minimal environmental risk. Future research should delve deeper into the mechanisms of selectivity of piplartine, evaluating its effectiveness in different ecological contexts and its long-term safety, especially under natural conditions. It is also important to investigate the interaction of piplartine with other substances present in aquatic ecosystems and its effects on organisms at different trophic levels. This will enable the development of more sustainable and effective strategies for vector control, minimizing environmental impacts and promoting biodiversity conservation.

## Figures and Tables

**Table 1:** NMR data of piplartine (500 MHz, CD_3_OD, δ ppm) and comparison with literature data.

Position	*δ*_H_ (*J* in Hz)	*δ*_H_ (*J* in Hz) ^a^
**2**	-	-
**3**	6.01 (*dt*, 9.8, 1.8)	6.03 (*dt*, 9.7, 1.6)
**4**	7.08 (*dt*, 9.8; 4.3)	6.94 (*dt*, 9.7; 4.1)
**5**	2.51 (*tdd*, 6.6; 4.3; 1.8)	2.47 (*m*)
**6**	3.99 (*t*, 6.6)	4.03 (*t*, 6.4)
**7**	-	-
**8**	7.38 (*d*, 15.9)	7.41 (*d*, 15.6)
**9**	7.61 (*d*, 15.9)	7.66 (*d*, 15.6)
**10**	-	-
**11**	6.92 (*s*)	6.79 (*s*)
**12**	-	-
**13**	-	-
**14**	-	-
**15**	6.92 (*s*)	6.79 (*s*)
**16**	3.88 (*s*)	3.88 (*s*)
**17**	3.80 (*s*)	3.86 (*s*)
**18**	3.88 (*s*)	3.88 (*s*)

^a^ [30,31,32] (1H em CDCl3, 500 MHz).

**Table 2:** Estimated lethal concentrations of piplartine and *α*-cypermethrin against *Ae. aegypti* and *An. darlingi* larvae.

Sample	Larvae	LC_50_ (μg/mL) (LCL-UCL)	LC_90_ (μg/mL) (LCL-UCL)	χ^2^ (Df)	Linear Equation	Relative Potency
Piplartine	*Ae. aegypt*	14.56 ^b^ (13.742- 15.364)	23.80 ^b^ (22.243- 25.871)	5.272 (4) *	Y = −6.992 + 2.610	0.0017
	*An. darlingi*	16.49 ^c^ (15.642–17.332)	26.44 ^b^ (24.716–28.771)	4.004 (4) *	Y = −7.611 + 6.252	0.0013
*α*-Cypermethrin	*Ae. aegypt*	0.023 ^a^ (0.021–0.025)	0.049 ^a^ (0.045–0.056)	8.091 (4) *	Y = −6.386 + 3.908	1
	*An. darlingi*	0.025 ^a^ (0.023–0.026)	0.043 ^a^ (0.040–0.048)	3.112 (4) *	Y = −8.429 + 5.250	1

LC_50_ and LC_90_—lethal concentrations to kill 50 and 90% of larvae. LCL—lower confidence limit of 95%. UCL—upper confidence limit of 95%. * Non-significant chi-squared (*p* > 0.05). Df—degree of freedom. Letters (a–c) within the same column indicate statistical differences, as determined by ANOVA (one-way) and Tukey’s test. For LC_50_, the results were F(3, 8) = 705.8, *p* < 0.0001 and for LC_90_, F(3, 8) = 344.3, *p* < 0.0001. RP—relative potency = LC_50_ of standard/LC_50_ of botanical larvicidal [36].

**Table 3:** Cox regression analysis investigating the influence of treatments and order as covariates on survival outcome.

Covariates	Coefficient (B)	Exp(B) * (Odds Ratio)	Confidence Interval (95%)	*p*-Value
Piplartine	−0.001	0.999	0.500 to 1.500	1.000
DMSO	−0.001	0.999	0.500 to 1.500	1.000
*α*-Cypermethrin	4.000	54.598	1.500 to 199.700	0.030
Order	0.005	1.005	0.811 to 1.245	0.964

* Exponential of the coefficient B.

## Data Availability

The datasets generated during and analyzed during the current study are available from the corresponding author upon reasonable request.

## Supplementary Materials

The following supporting information can be downloaded at: https://www.mdpi.com/article/10.3390/plants14050774/s1, Figure S1.
