# Supplementary material for: Bioassay-Guided Isolation of Piplartine from Piper purusanum Yunck (Piperaceae) and Evaluation of Its Toxicity Against Aedes aegypti Linnaeus, 1762, Anopheles darlingi Root, 1926 (Culicidae), and Non-Target Animals
Source: Plants (Basel). 2025 Mar 3;14(5):774. doi: 10.3390/plants14050774 (PMC11901690; doi:10.3390/plants14050774)
Supplement: Supplementary file 1 [file plants-14-00774-s001.zip › plants-3480072-supplementary.pdf]

Supplementary Information

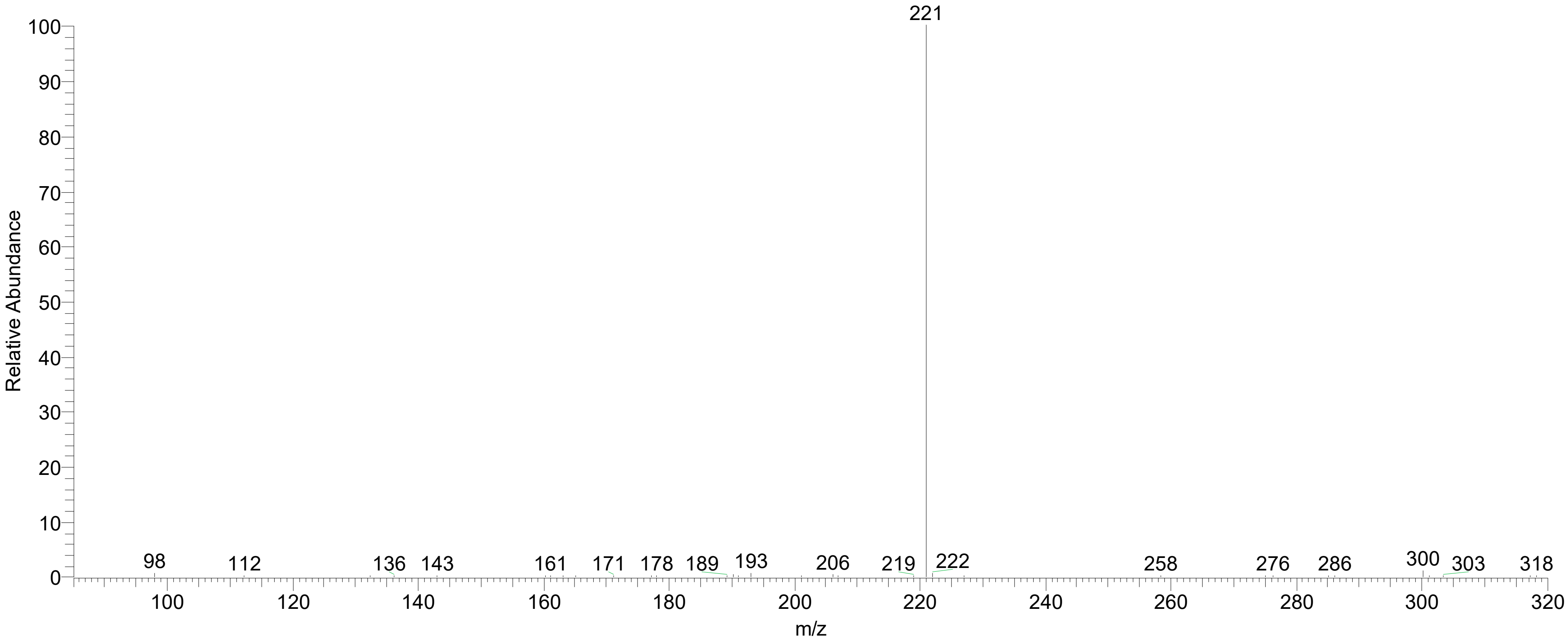

Figure S1. APCI-MS/MS spectrum of the piplartine.

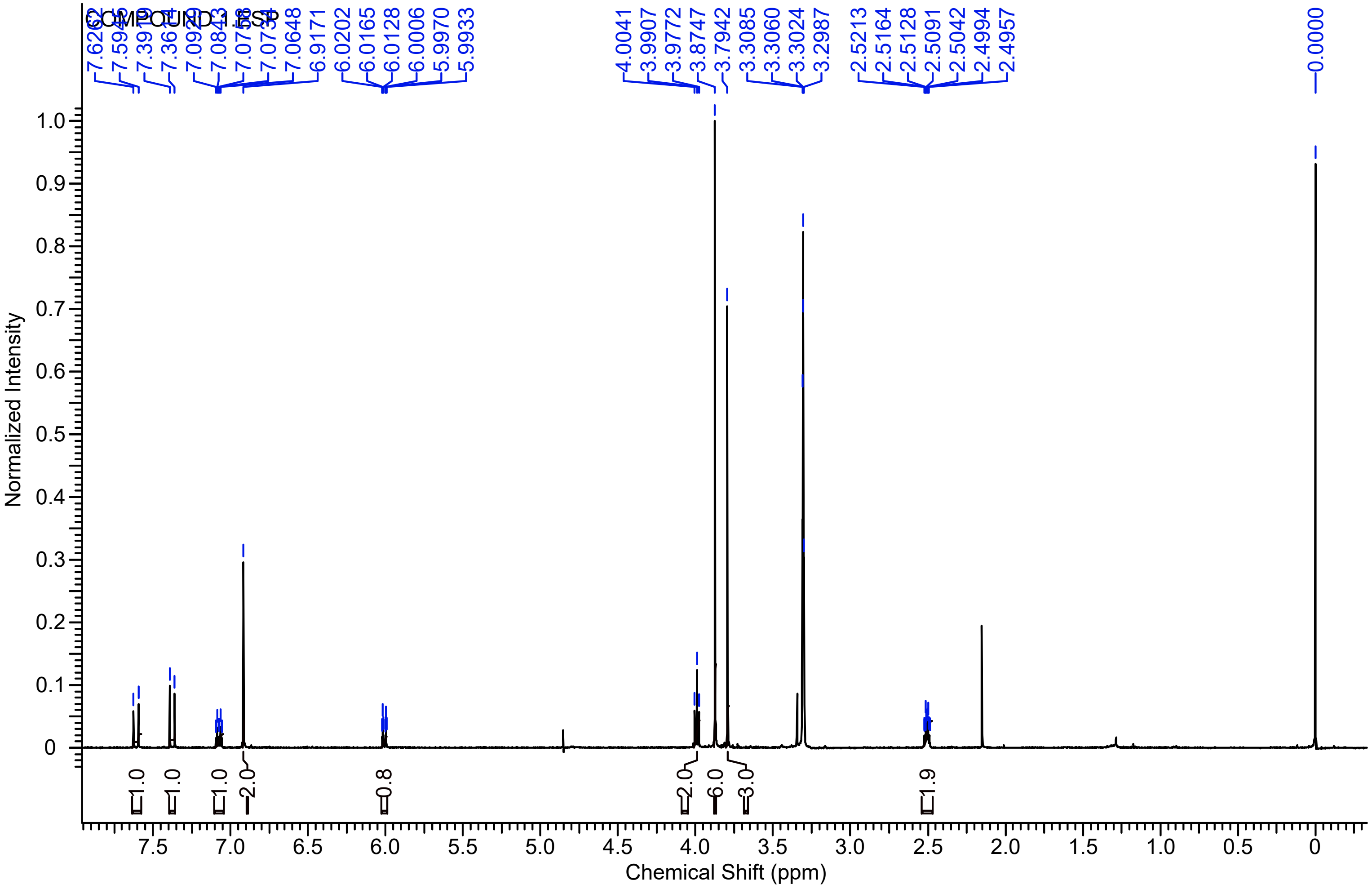

Figure S2. ^{1}H NMR spectrum (500 MHz, CD_3OD) of the piplartine.

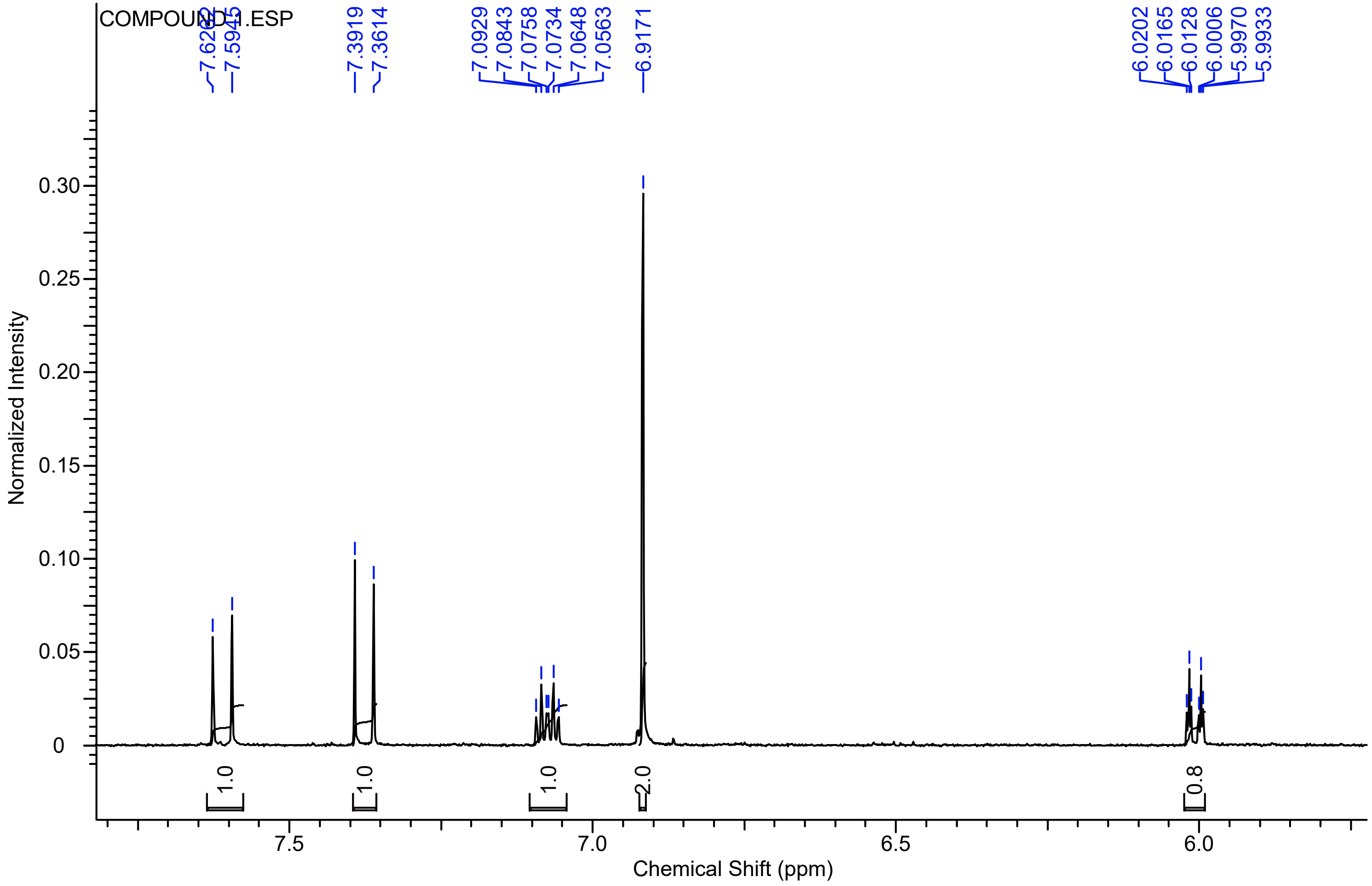

Figure S3. ^{1}H NMR spectrum (500 MHz, CD_3OD) of the piplartine.

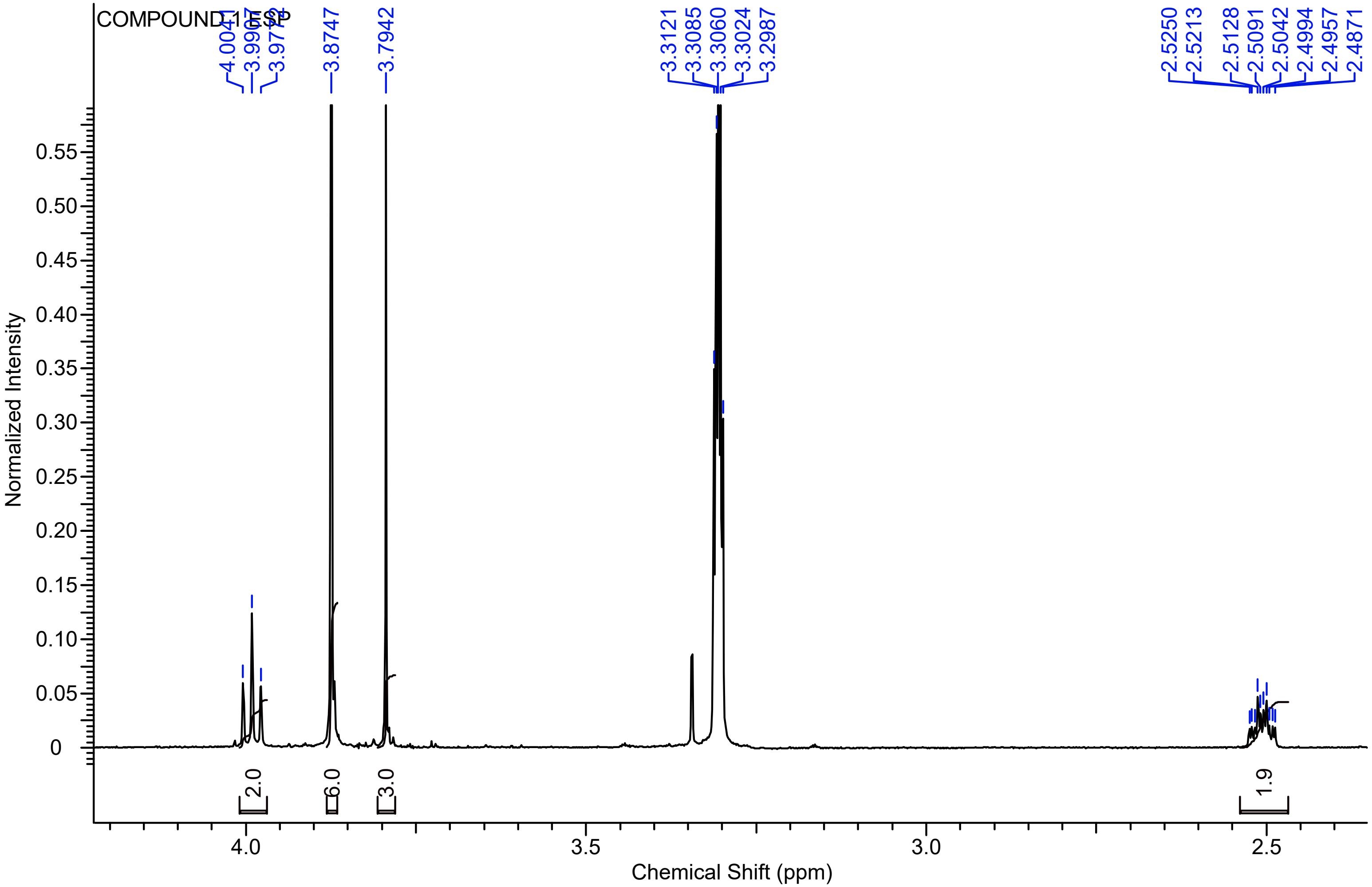

Figure S4. ^{1}H NMR spectrum (500 MHz, CD_3OD) of the piplartine.
